# Mismatch repair is a double-edged sword in the battle against microsatellite instability

**DOI:** 10.1017/erm.2022.16

**Published:** 2022-09-05

**Authors:** Carson J. Miller, Karen Usdin

**Affiliations:** Laboratory of Cell and Molecular Biology, National Institute of Diabetes, Digestive and Kidney Diseases, National Institutes of Health, Bethesda, MD, USA

**Keywords:** Mismatch Repair (MMR), Repeat expansion Diseases, Microsatellite instability (MSI), Short tandem repeats (STRs)

## Abstract

Roughly 3% of the human genome consists of microsatellites or tracts of short tandem repeats (STRs). These STRs are often unstable, undergoing high-frequency expansions (increases) or contractions (decreases) in the number of repeat units. Some microsatellite instability (MSI) is seen at multiple STRs within a single cell and is associated with certain types of cancer. A second form of MSI is characterised by expansion of a single gene-specific STR and such expansions are responsible for a group of 40+ human genetic disorders known as the repeat expansion diseases (REDs). While the mismatch repair (MMR) pathway prevents genome-wide MSI, emerging evidence suggests that some MMR factors are directly involved in generating expansions in the REDs. Thus, MMR suppresses some forms of expansion while some MMR factors promote expansion in other contexts. This review will cover what is known about the paradoxical effect of MMR on microsatellite expansion in mammalian cells.

## Introduction

Tracts containing tandem arrays of short perfect- or near-perfect repeat units are common in the human genome (Ref. 1). These short tandem repeats (STRs), or microsatellites, consist of repeat units that are generally ~1–6 nucleotides long. The STRs are found in promoters, exons, introns, as well as in intergenic regions where they can impact gene expression in a myriad of different ways, including affecting promoter activity, RNA polymerase processivity, splicing, translation rates and protein function (Ref. 2). Many STRs are polymorphic, giving rise to expansions, or increases in the number of repeat units; as well as contractions, or loss of repeat units. Such STRs are sometimes referred to as variable number tandem repeats (VNTRs). STRs can be a significant source of human genetic variation and the instability of some of these tracts can have biological consequences because of their intrinsic effects on gene expression (Refs 2, 3). In addition, many of these sequences form secondary structures that are thought to make them difficult to replicate (Refs 4–11). This can result in the generation of chromosome abnormalities of different kinds (reviewed in Ref. 12).

Two major classes of human STR expansions are known: the first class is associated with genome-wide microsatellite instability (MSI), while the second class is associated with expansions of a specific microsatellite. Genome-wide MSI is associated with a predisposition to certain cancers including haematological malignancies as well as certain colon, urothelial, hepatobiliary, pancreatic, bladder, kidney, prostate, endometrial, ovarian and breast cancers (Refs 13–20). In contrast, the locus-specific expansions define the repeat expansion disorders (REDs), a group of 40+ human genetic disorders that are primarily neurological or neurodevelopmental in nature. Diseases in this group include Huntington's disease (HD), caused by expansion of CAG/CTG-STR in the first exon of the huntingtin (*HTT*) gene (Ref. 21); Friedreich ataxia (FRDA), caused by a GAA/TTC-STR in intron 1 of the frataxin (*FXN*) gene (Ref. 22); myotonic dystrophy type 1 (DM1), caused by expansion of a CTG/CAG-STR in the 3′ UTR of the *DMPK* gene (Refs 23, 24); and the Fragile X-related disorders (FXDs), resulting from expansion of a CGG/CCG-STR in the 5′ UTR of the *FMR1* gene (Refs 25–28). While it was initially thought that STR expansions in the REDs occurred by a mechanism similar to cancer-associated MSI, emerging evidence suggests these two types of STR expansions have completely different molecular mechanisms. Genome-wide MSI results from errors arising during DNA synthesis that normally would be repaired by the MMR machinery, that is, MMR factors all act anti-mutagenically at these loci to suppress expansions. In contrast, STR expansions in the REDs actually require certain components of the MMR machinery, that is, these MMR factors can also act pro-mutagenically. While work in model systems suggests that other mechanisms of STR expansion may be possible (e.g. Ref. 29), this review will focus on what is currently known about STR expansions arising from either the pro- or anti-mutagenic roles of MMR.

## Cancer-associated MSI

**Fig. 1.**
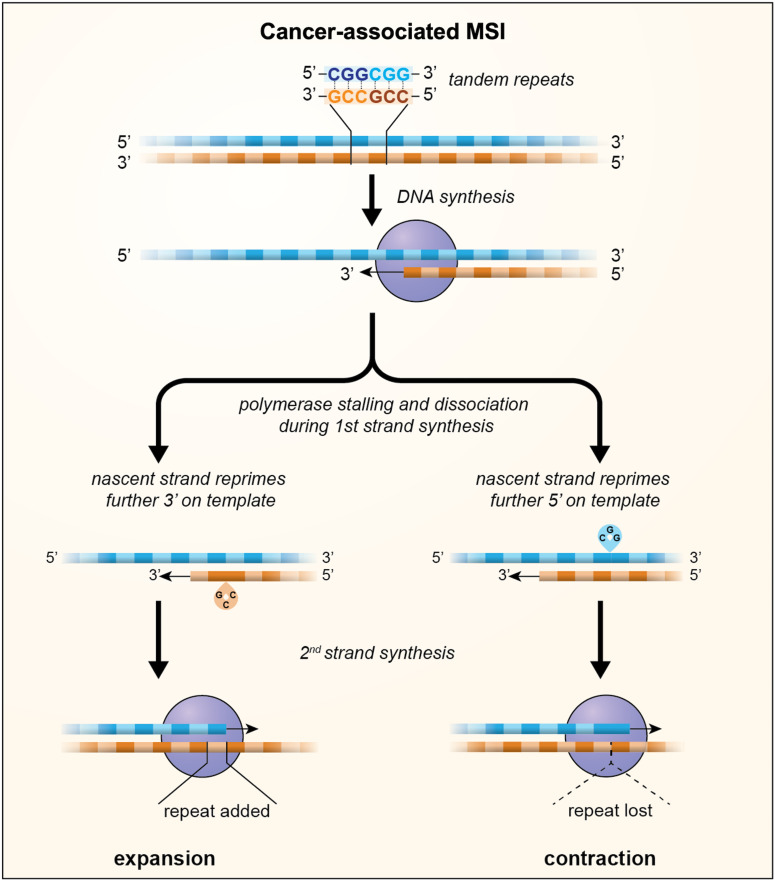
A model for MSI in which loop outs generated by strand-slippage of the nascent strand during DNA replication escape MMR and result in the incorporation of a small number of additional units, indicated by the green boxes, or the loss of a small number of repeat units, indicated by the dotted triangle. Whether repeats are gained or lost depends on whether repriming occurs further 3′ on the template resulting in nascent strand loop outs or further 5′ on the template resulting in loop outs being formed on the template strand.

During DNA replication two types of errors can be introduced into DNA: mismatches and insertions or deletions (INDELs). Mismatches arise from insertion of the incorrect base in the daughter strand by the DNA polymerase. Most of these mismatches are removed by the proofreading function of the polymerase, but those that escape this proofreading will cause point mutations if the daughter strand is replicated before the mismatch is removed (Refs 30, 31). INDELs are thought to result primarily from the dissociation of the DNA polymerase from the template thus creating an opportunity for two strands of DNA to slip relative to one another. Dissociation might be exacerbated by an encounter with impediments to replication fork progression such as those formed by unusual DNA structures, strongly bound proteins or collisions with a transcription complex, while mispriming may be favoured when one of the DNA strands forms a stable secondary structure (Ref. 32). When strand-slippage occurs within a STR, out-of-register reannealing can occur with priming from the slipped position as illustrated in Figure 1. This results in either a loop out of the template strand or a loop out on the nascent strand depending on whether reannealing occurs 5′ or 3′ on the template. Failure to remove the loop out leads to expansions if the loop out is in the nascent strand and contractions if it is in the template strand (Refs 33–36).

Repair of these replication errors is carried out primarily by the MMR machinery that travels behind the replication complex. In eukaryotes, recognition of mismatches and INDELs during MMR is accomplished by either of two MutS complexes, both of which are heterodimers of homologous proteins: MSH2/MSH6 in the MutS*α* complex and MSH2/MSH3 in the MutS*β* complex. MutS*α* is primarily involved in the recognition of mismatches and 1 base INDELs (Refs 37–39). MutS*β*, on the other hand, is involved primarily in the repair of larger loops (Refs 40–42). (A third MutS complex found in mammals, MutS*γ*, is a MSH4/MSH5 heterodimer that functions almost exclusively in meiotic crossover resolution (Ref. 43).) After lesion binding, MutS*α* and MutS*β* recruit a member of the MutL family of proteins. Mammals have three different MutL complexes. Like the MutS complexes, each of the MutL complexes are heterodimers; consisting of MLH1 bound to either PMS2, MLH3 or PMS1 to form MutL*α*, MutL*γ* or MutL*β*, respectively (Refs 44–46). MutL*α* is the most abundant of the MutL complexes and is responsible for most MMR. MutL*γ* plays a minor role in MMR, primarily in cooperation with MutS*β* (Refs 47, 48). MutL*α* and MutL*γ* are nucleases that introduce nicks into the MMR template, a critical step in the repair process. The role of MutL*β* in MMR is unclear (Refs 49, 50). While PMS1 is more abundant than MLH3, it lacks the DQHA(X)_2_E(X)_4_E nuclease motif present in both PMS2 and MLH3 and its loss is not associated with increased MSI in mice (Ref. 49). Multiple lines of evidence suggest that after initial mismatch binding by MutS*α*, additional MutS*α* complexes load onto the DNA, followed by recruitment of multiple MutL*α* proteins (Refs 51, 52). A similar situation may apply to MutS*β*-directed recruitment of MutL*γ*, since formation of MutL*γ* polymers on the mismatch template has also been shown to be important for proper MutL*γ*-mediated repair (Ref. 53). Excision of the nicked strand is carried out by a 5′ to 3′ exonuclease such as exonuclease 1 (EXO1) (Refs 54, 55) or FAN1 (Ref. 56). Strand-displacement synthesis by Pol *δ* can also remove the nicked strand. This is followed by repair synthesis by Pol *δ*, with sealing of the remaining nick by DNA ligase I to complete the repair process.

While this process is relatively efficient, strand-slippage occurs so frequently that some MSI occurs even in the presence of the normal MMR machinery. Mean rates of ~10^−5^ to 10^−7^ MSI events per locus per cell generation have been reported in human cells with functional MMR, orders of magnitude higher than the mutation rate seen in unique sequence (Refs 57–59). Loss of MMR results in rates of MSI that can be 2–3 orders of magnitude higher (Ref. 60). The wide variation in mutation rates of different STRs is related in part to the size of the repeat units, their sequence composition and the size and purity of the repeat tract (Refs 61, 62). The likelihood of instability at a specific microsatellite is also related to the normal target of the dysfunctional MMR gene. So, mutations in MSH2, MLH1 and PMS2 increase instability of microsatellites containing mononucleotide, dinucleotide and tetranucleotide repeat units; MSH6 mutations affect microsatellites with mononucleotide and some dinucleotide repeat units; and MSH3 mutations affect dinucleotide and tetranucleotide containing repeat units, but not ones consisting of mononucleotide repeat units.

More than 90% of MSI events involve the gain or loss of a single repeat unit with a very limited number of mutations involving multiple units (Ref. 63). MSI often exhibits an expansion bias (Refs 64–67). This bias is reduced at very large microsatellites (Refs 64, 65), perhaps reflecting the formation of stable secondary structures and the resultant difficulties associated with replication of the region. This could result in a dependency on proteins such as the Werner's syndrome helicase (WRN) to remove the secondary structure thus allowing replication to proceed (Ref. 68). It has also been suggested that these structures promote error-prone DNA synthesis resulting in mutations that affect the purity of the repeat tract (Ref. 10). This in turn would reduce the likelihood of further expansion. While most MSI events of this kind involve a single repeat unit, MSI with an expansion bias could over time result in the large microsatellites that accumulate in cancer cell lines such as HCT116 and KM12 that lack MLH1 (as well as MSH3 in the case of HCT116) (Ref. 68).

## MSI in the REDs

In contrast, studies in RED patient cohorts using genome-wide association (GWA) or the testing of candidate MMR gene polymorphisms suggest that functional MMR components are required for some, if not all, STR expansions that cause the REDs (Refs 69–74). This is consistent with evidence from mouse and human cell models of a number of these disorders that shows a requirement for MutS*β* and MutL*γ* (reviewed in Ref. 75). Canonical MMR *per se* is unlikely to be responsible for these expansions since mutations in EXO1 and FAN1, 5′-3′ exonucleases that act downstream of the MutS and MutL proteins in normal MMR, protect against expansion in mouse or human tissue culture models (Refs 73, 76–79), and GWA studies data are consistent with a protective role for FAN1 in reducing expansions in humans (Refs 69, 73). Furthermore, Lig4, the ligase required for non-homologous end joining (NHEJ), a form of double-strand break repair (DSBR), also protects against expansion in a mouse model of the FXDs (Ref. 80). This suggests that NHEJ competes with the expansion pathway for access to a common DSB intermediate.

As with cancer-associated MSI, the extent of expansion in the REDs is related in part to the length and purity of the repeat tract (Refs 81–88). Mathematical modelling of human somatic expansions and empirical observations of both germline and somatic expansions over time in mice support the idea that most expansion events involve the addition of 1–2 repeat units (Refs 89, 90). As with expansions arising in MMR-deficient cells, this can result in the production of much larger alleles over time. However, in some cell types the MMR factor-dependent expansions occur at frequencies that are orders of magnitude higher than the MSI occurring in the absence of MMR at the same locus (Ref. 91).

The mechanism responsible for this high-frequency expansion process is not fully understood. Clues to what this process may be include the fact that expansions can occur in post-mitotic cells such as oocytes and neurons (Refs 78, 92–95). Thus, these events can be independent of chromosomal replication. Furthermore, the fact that the STR in the X-linked *FMR1* gene that causes the FXDs only expands when it is on the active X chromosome indicates that STR expansion requires transcription or transcriptionally-competent chromatin (Ref. 96). A role for oxidative damage is suggested by the fact that the loss of OGG1 and NEIL1, DNA glycosylases involved in the base excision repair of oxidative damage, decreases the expansion frequency in a HD mouse model (Refs 97, 98) and exogenous sources of oxidative stress increase the expansion frequency in some mouse and tissue culture models (Refs 99, 100). However, antioxidants only have a minimal effect on expansion (Refs 101, 102) and even in the absence of OGG1 and NEIL1 many expansions are still seen (Refs 97, 98). Thus, endogenous oxidative stress may not be the only trigger for the expansion process or even the most important one.

**Fig. 2.**
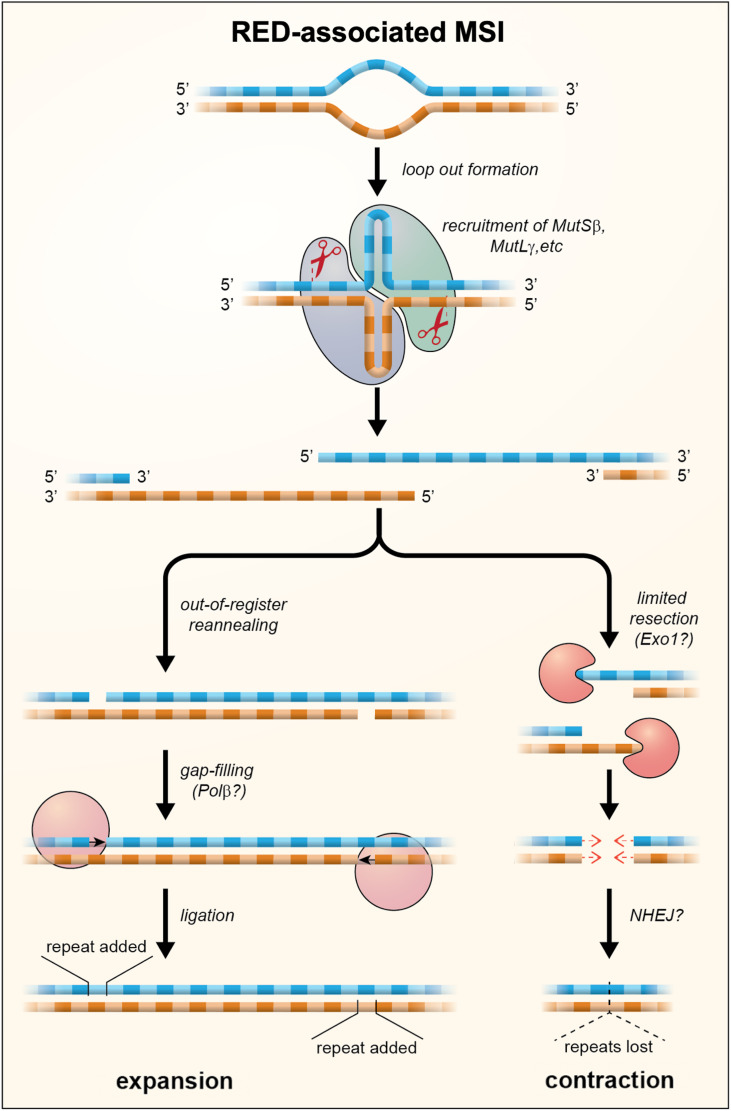
A model for repeat expansions and contraction in the REDs in which loop outs are formed on one or both strands during transcription or at other times that the DNA was unpaired. These loop outs are then bound by MutS and MutL proteins. Cleavage by MutL*γ* results in the formation of a staggered DSB with 5′ overhangs. Out-of-register reannealing of the DSB can produce a substrate for simple gap filling which results in the addition of repeat units. Exonucleolytic processing of the DSB can result in products with shorter 5′ overhangs or blunt ends. These products may then be processed, perhaps by NHEJ or gap-filling, to generate the loss of repeat units as indicated by the dotted triangle. The extent of contraction would depend on the amount of exonucleolytic cleavage that occurs prior to repair.

Although MutL*γ* is the least abundant of all the MutL complexes, its nuclease activity is required for repeat expansion (Refs 103–105). Thus, expansion either involves a substrate that is bound preferentially by MutS*β*/MutL*γ* or MutL*γ* cleavage is uniquely able to generate an intermediate that can be processed to generate an expansion. Interestingly, MutL*γ* has been shown to cut the DNA strand opposite to the mismatch in vitro (Ref. 106) and MutL*γ* is required during meiosis for the resolution of Holliday junctions (HJs) (Ref. 107). Loop outs formed within the STR by both DNA strands might resemble such a four-way junction. Such structures could potentially arise any time the repeat tract was unpaired since out-of-register reannealing could occur particularly if one or both strands formed stable secondary structures as many STRs do (reviewed in Refs 75, 108). Cleavage of the opposite strand at each of the loop outs could then result in a staggered DSB. Interestingly, we have shown that EXO1, which plays a structural role in determining the orientation of cleavage of HJs (Ref. 47), also plays a nuclease-independent role in protecting against repeat expansion (Ref. 77). This raises the possibility that cleavage of the expansion intermediate may result in a DSB that is prone to expand and one that is not. A model for repeat expansion that accommodates these observations is shown in Figure 2. In this model expansions arise when out-of-register reannealing of the DSB occurs. This leaves a gap of a small number of repeat units that is then repaired by gap-filling. The net effect is that a small number of repeats are added to the repaired allele.

In addition to MutS*β* and MutL*γ*, MutL*β* has also been shown to be required for expansion in embryonic stem cells from a mouse FXD model (Ref. 87). An active role for MutL*β* in generating expansions in HD is also suggested by the fact that *PMS1* variants predicted to be deleterious are most frequently associated with a later age at onset/less severe phenotype in HD cohorts (Ref. 73). However, since MutL*β* lacks a nuclease and is not required for MMR, how it contributes to expansions is unclear. This is not the only unresolved issue; although MutS*α* contributes to expansions in FXD and FRDA mice and FRDA iPSCs (Refs 31, 109, 110), little if any effect of the loss of MSH6 was seen in DM1 mouse model (Ref. 111) or in a human cell model system of FRDA (Ref. 112). Furthermore, reducing MutL*α* levels also has different effects in various systems. In a mouse model of the FXDs, MutL*α* is required for expansions in ESCs (Ref. 87), while in a mouse model of DM1 loss of MutL*α* only resulted in a 50% decrease in expansions (Ref. 113). Reduced PMS2 caused no change in the expansion frequency in a human cell model of FRDA (Ref. 100), whereas a mouse model of FRDA lacking *Pms2* showed an increase in expansions (Ref. 109). The latter is consistent with the observation that a missense mutation in *PMS2* correlates with an earlier age at onset in HD (Ref. 69). These differences do not necessarily mean that the expansion mechanisms in these diseases are fundamentally different. Since multiple MutS and MutL complexes are involved in binding to a mismatch, a case can be made that MutS*α* and MutL*α* are able to act in an auxiliary capacity to promote expansions when the essential factors, such as MutS*β* and MutL*γ*, and perhaps MutL*β*, are limiting (Ref. 75).

Contractions are also seen in the REDs-associated STRs and their mouse models, although their aetiology is less well understood. A bimodal distribution of contractions is seen in the germline of a mouse model of the FXDs, with some contractions involving the loss of just 1–2 repeat units whilst others involve the loss of much larger numbers of repeat units (Ref. 114). These larger contractions are sometimes difficult to discern in somatic cells because, unlike expansions, the contraction products do not seem to fall into a single size class. Curiously, loss of MSH3 results in a decrease not only in expansions, but also in the number of large contractions that are observed, with the decrease in these events being associated with a corresponding increase in the number of small contractions (Ref. 114). One model consistent with these observations is that large contractions represent a second possible outcome of the events that give rise to expansions, with contractions arising from DSBs that undergo some exonucleolytic cleavage prior to DSBR as illustrated in Figure 2.

## Concluding remarks

Thus, MMR factors can both suppress and promote MSI. Which MSI pathway predominates is likely to depend on a variety of cell-type specific factors including the frequency of cell division and the relative levels of factors that promote or suppress each type of MSI. For example, while MMR is important for preventing genome-wide MSI in the colon, as evidenced by the high frequency of MSI-high colonic tumours in individuals with germline mutations in MMR proteins, MSI-high tumours originating in neurons are rare (Refs 115–117). The low level of MSI-high tumours in neurons may reflect in part the fact that neurons are non-dividing and thus likely to rarely generate the substrates for the MMR pathway. The high tumour incidence in the colon might reflect the consequences of exposure to dietary mutagens in rapidly dividing cells. In contrast, repeat expansion in neurons, particularly striatal neurons, occurs at high frequency in both mouse models of REDs and in REDs patients (Refs 118–120). The high frequency of STR expansion in neurons of REDs patients may reflect the high levels of factors such as MutS*β*, OGG1 and NEIL1 that promote expansion, and low levels of proteins such as EXO1, that protect against them (Ref. 77). The high levels of transcription of the affected genes in neurons may also contribute to the incidence of these expansions, by increasing the opportunity for formation of the substrates upon which the expansion process acts.

The paradoxical effect of MMR proteins on MSI is particularly apparent at the *Fmr1* locus in embryonic stem cells from a mouse model of the FXDs. Consistent with the role of functional MMR components in generating expansions, a high rate of expansions is seen at this locus in MMR-proficient cells derived from these mice. For example, most alleles with ~280 repeat units gained an additional 19 repeats over a 52-day period in cells wildtype for MLH3 (Ref. 87). In contrast, in cells with a similar repeat number that lacked MLH3, the modal size of the allele actually decreased by one repeat over the same period. Thus, the same microsatellite expands rapidly in MMR-proficient cells and contracts more slowly in MMR-deficient ones. The fact that MMR-deficiency results in contractions at this locus rather than expansions serves to emphasise the fundamentally different events occurring at this locus.

In the case of MSI-high tumours, the expanded microsatellites themselves might be a source of vulnerability that could be exploited for therapeutic purposes. Since expansion results in a dependence on DNA helicases such as WRN (Ref. 68), it may be possible to selectively eliminate the cancer cells using a synthetic lethal approach that targets these enzymes (Ref. 121). In the case of many REDs, a growing body of evidence suggests that somatic expansion of the disease-associated STR significantly worsens the age at onset and/or disease severity (Refs 69, 70, 72, 73). Since most of these diseases are severely life-limiting and lack any effective treatment or cure, there is an interest in exploring ways to reduce this MSI in somatic cells. This approach has additional appeal in that any success in this regard would be relevant to multiple REDs. Of course, given the requirement of many MMR factors for protecting against cancer, targeting these factors to reduce expansion poses a challenge. However, MSH3 and MLH3 are not major players in MMR and may thus be acceptable targets particularly for those diseases with a high early mortality. For example, tail vein injection of a splice switching oligonucleotide that favours the production of an MLH3 isoform lacking the nuclease domain has already been shown to reduce expansion in some peripheral tissues of a mouse model of HD (Ref. 101). Since the absence of PMS1 is not associated with tumour susceptibility or any other obvious phenotype in mice (Ref. 49), depletion of this factor may be an even more attractive approach. While delivery of therapeutics to deep brain regions such as the striatum is not trivial, implanted intracerebroventricular devices have been used successfully for decades to deliver chemotherapeutic agents to treat central nervous system malignancies (Ref. 122). This experience could perhaps be leveraged for the ongoing delivery of MMR-targeting molecules to treat REDs.
